# Rare post-operative intracranial abscess due to *Serratia marcescens*: what we can learn from it?

**DOI:** 10.1186/s12879-023-08966-7

**Published:** 2024-01-08

**Authors:** Wenzheng Liu, Ridong Feng, Xiaolin Song, Hai Zhao

**Affiliations:** 1 Department of Neurology, the Affiliated Hospital of Qingdao University, No. 16 Jiangsu Road, Qingdao, Shandong 266005 China; 2 Department of Neurosurgery, the First Affiliated Hospital of Zhejiang University, Hangzhou, 310003 China; 3 Department of Neurosurgery, the Affiliated Hospital of Qingdao University, No. 16 Jiangsu Road, Qingdao, Shandong 266005 China

**Keywords:** Intracranial abscess, Serratia marcescens, Titanium mesh, Hair removal, Antibiotics

## Abstract

**Background:**

Nosocomial infections caused by Serratia marcescens mostly occurred in pediatrics and it was very rarely reported after adult surgery. Here, an intracranial abscess caused by *Serratia marcescens* was reported.

**Case summary:**

We report a rare case of a postoperative intracranial abscess caused by *Serratia marcescens* in a 63-year-old male patient with a left parietal mass. The patient underwent resection of the mass on June 1, 2022, and the postoperative pathology revealed an angiomatous meningioma, WHO I. He then experienced recurrent worsening of right limb movements, and repeated cranial CT scans showed oozing blood and obvious low-density shadows around the operation area. Delayed wound healing was considered. Subsequently, a large amount of pus was extracted from the wound. The etiological test showed that *Serratia marcescens* infection occurred before the removal of the artificial titanium mesh. Antibiotics were initiated based on the results of drug susceptibility tests. At present, the patient is recovering well and is still closely monitored during follow-up.

**Conclusion:**

It is rare for *Serratia marcescens* to cause brain abscesses without any obvious signs of infection. This report provided in detail our experience of a warning postoperative asymptomatic brain abscess caused by an uncommon pathogen.

## Introduction

Bacterial infections of the central nervous system (CNS) remain to be an important cause of morbidity and mortality after craniotomies. ﻿The incidence rate ﻿is 1–3%, with *Staphylococcus aureus* *(S. aureus)* being the most common pathogen. *Serratia marcescens* is a gram-negative enteric bacillus that rarely colonizes human hosts. It ﻿is widely distributed in hospital environments and is regarded as an opportunistic pathogen that occasionally causes various infections, such as wound infections and pneumonia [1–4]. Most brain abscesses caused by *Serratia marcescens* occurred in ﻿settings such as intensive care units (ICUs), especially neonatal units (NICUs) [5–9]. *Serratia marcescens*, ﻿a member of the *Enterobacteriaceae,* ﻿is a well-known pathogen of the respiratory tract, urinary tract, and a common etiology of wound infections, which has raised concerns as a causative pathogen of nosocomial infections [10, 11]. However, *Serratia marcescens* is a rare pathogen of adult CNS infections [12]. Here, we present a case of ﻿brain abscess caused by *S. marcescens*, with accompanying review of literatures.

## Case presentation

**Fig. 1:**
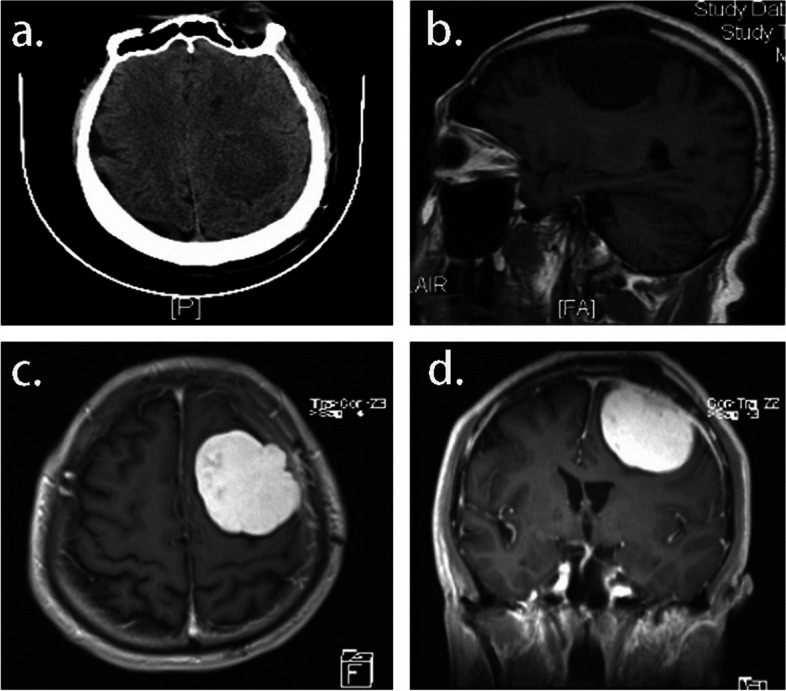
**A** Immediate preoperative CT performed at initial encounter demonstrated a minimally mass effect in the left parietal region. **B** Large well-defined mass lesion in the left posterior region with adjacent skull erosion was shown in sagittal T1. On axial (**C**) and coronal (**D**) enhanced T1, strong post-contrast enhancement was observed with dural tail sign, exerting mass effect on the left lateral ventricle with resultant shift of midline structures to the right

A 63-year-old man presented with complaints of right limb weakness over the past 4 months and was brought to the emergency department after a seizure episode. The patient had a 10-year history of unstable angina and underwent skin grafting 5 years prior. ﻿There had no recent history of fever, excessive sleepiness, or nuchal rigidity. Neurological and systemic examinations revealed grade 4 + on the right limb on manual muscle testing. Computed tomography (CT) of the brain showed a lesion occupying the left parietal lobe with a mild mass effect and a midline shift. Convex meningioma was presumptively diagnosed based on magnetic resonance imaging (MRI) results **(**Fig. 1).

**Fig. 2:**
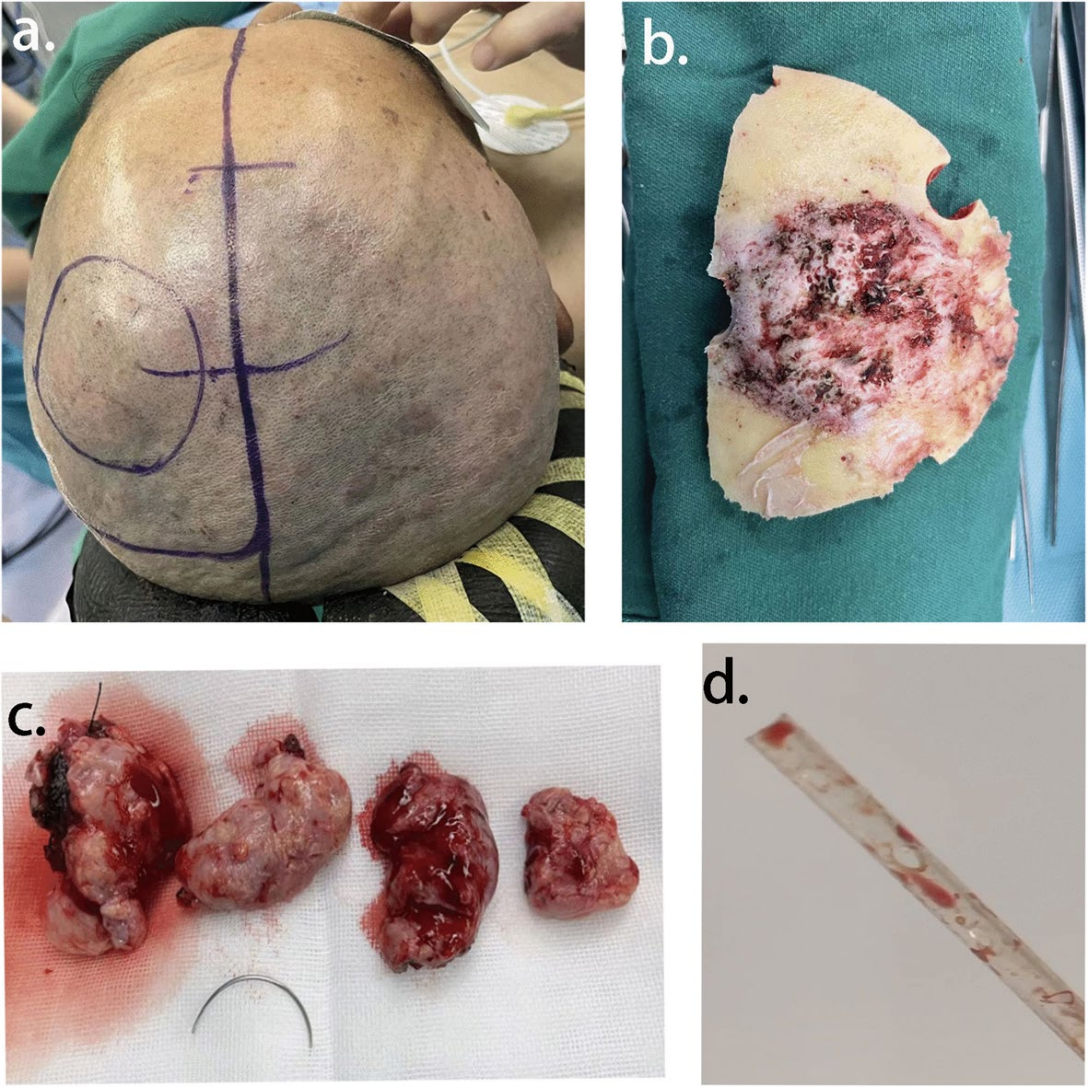
**A** Operative position, the hair was totally removed without obvious scalp injury. **B** In accordance with preoperative imaging data, skull erosion was found during craniotomy. **C** The operative specimen was removed in a piece meal fashion. **D** Prophylactic use of a subcutaneous closed suction drain was necessary for prevention of postoperative surgical site hematoma in craniotomy. Then, the drain was removed within 48 h after insertion without any apparent secretions

On June 1, 2022, the patient underwent left parietal craniotomy with total resection of the tumor. Consistent with Fig. 1B, the lesion exhibited bony erosion of the entire skull during craniotomy (Fig. 2). A histopathological examination of an intra-operative fast frozen section biopsy (Identification number D22-27096; Qingdao, China) demonstrated a meningeal epithelial tumor with mild-moderate cellular atypia and rich vessel supply, predisposed to atypical meningiomas (WHO II-III). It was recommended to wait for a large number of paraffin samples and immunohistochemistry (IHC) results to exclude the possibility of higher-grade lesions. The tumor had invaded the dura mater and skull. We performed an excision of the involved dura mater and skull, reaching a Simon Grade 5 level of resection. Consequently, in the process of cranial closure, we used an artificial dura mater for precise suturing of the dura mater and a titanium mesh to reconstruct the affected areas of the skull. This approach also potentially contributed to the risk of persistent and spreading infection in the patient. We sterilized the titanium mesh rigorously before it was implanted.

**Fig. 3:**
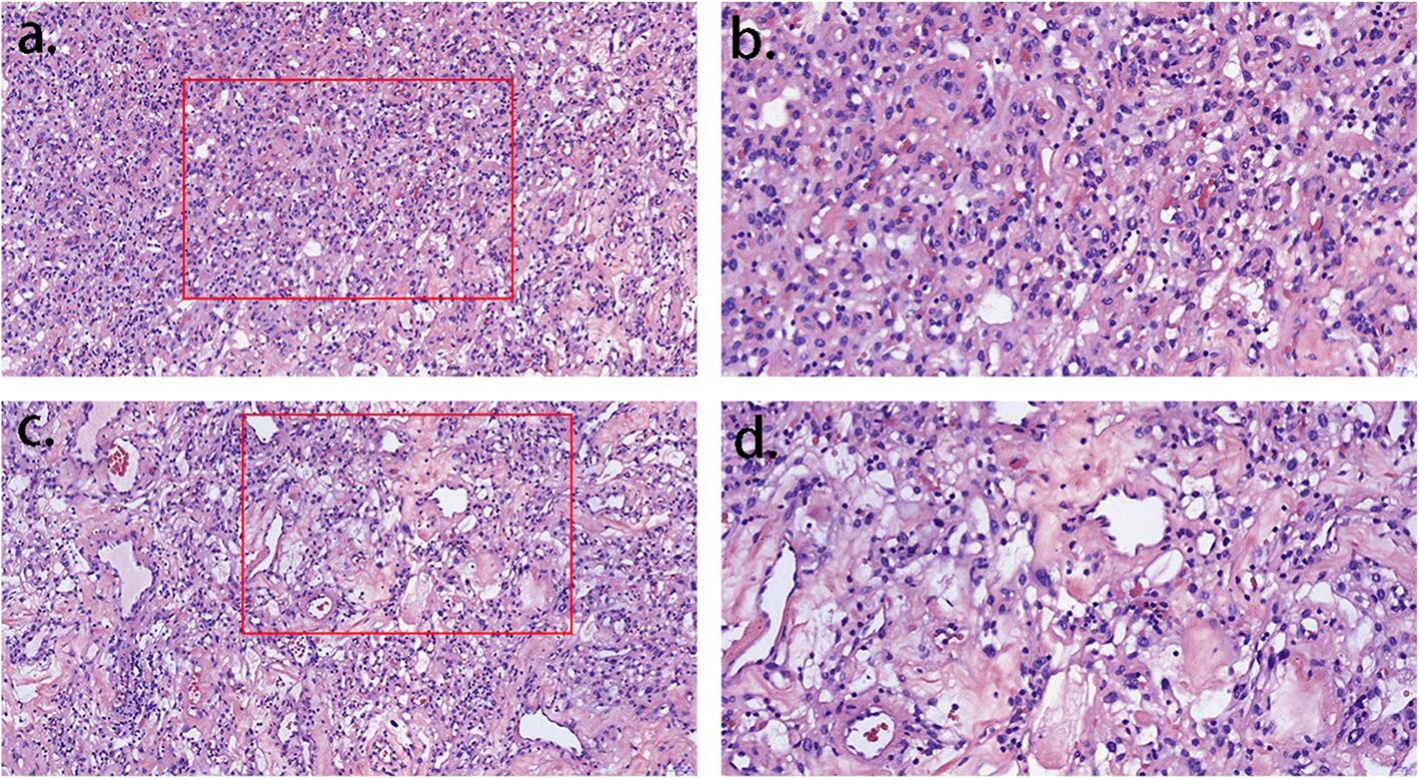
The final histological examination of the case confirmed marked hypervascularity with extensive hyalinization of both large and small vessels, along with the characteristics of angiomatous meningiomas

However, 8 days after the craniotomy, when all sutures were removed, the wound healed poorly. We changed dressings more frequently and, at the same time, applied vancomycin based on weight and creatinine clearance. Notably, the patient showed no signs of local and systemic infection. Thirteen days after craniotomy, the final histopathological diagnosis (Identification number D22-26985; Qingdao, China) confirmed the diagnosis of angiomatous meningioma, which was defined by hypervascularity, with tumoral blood vessels exceeding 50% of the total volume (WHO I) [13]. The IHC provided the definitive information –Vimentin ( +), SSTR2 ( +), PR ( +), CD34 ( +), STAT6 ( +), GFAP (-), S-100 (-), Olig-2 (-), Ki-67 (+ ,2%) (Fig. 3).

**Fig. 4:**
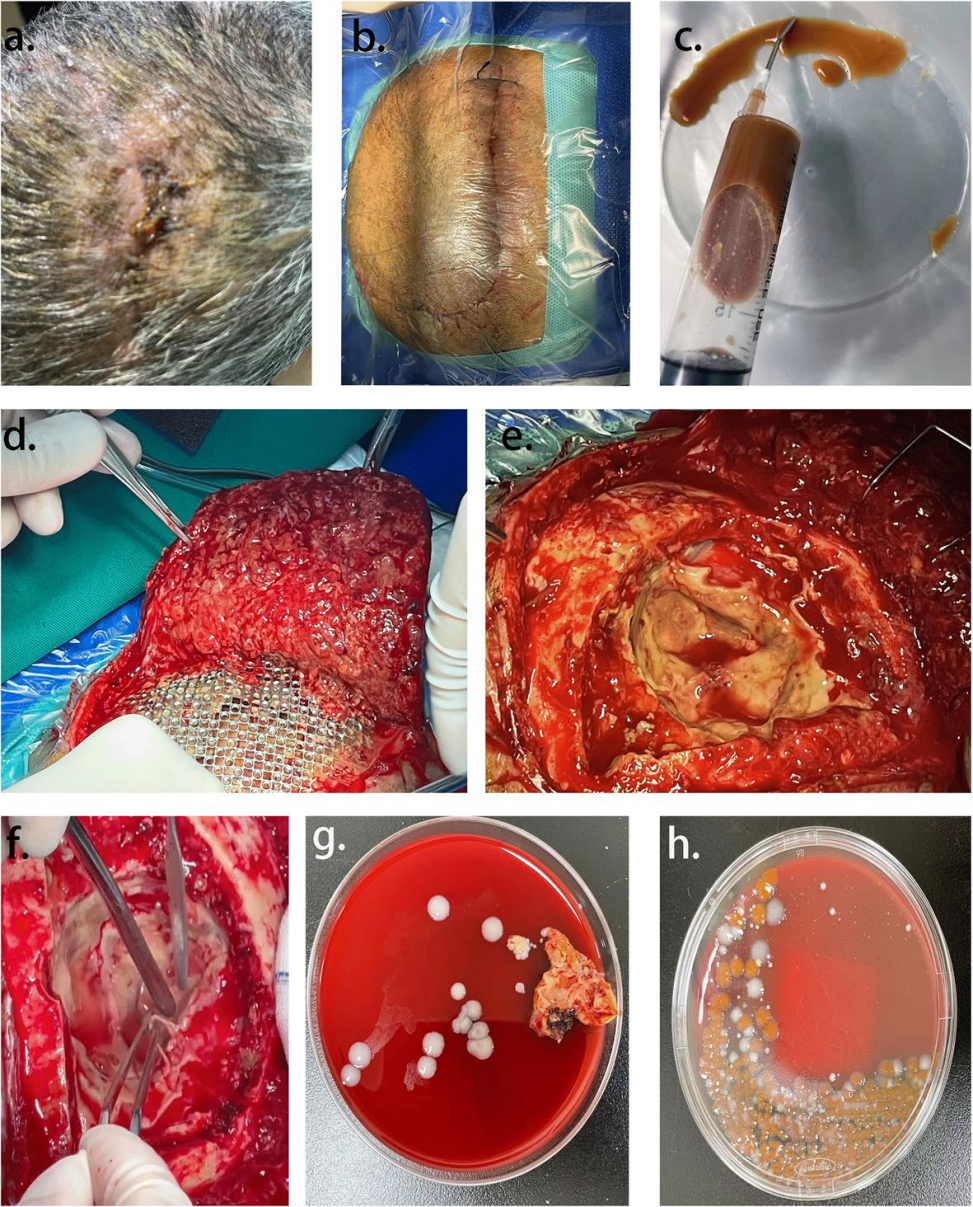
**A** Delayed wound healing was demonstrated. **B** In the same operative position, again the hair was totally removed. **C** Pus was removed from wound with sterile injection syringe. **D**-**F** Debridement of brain: First, artificial materials (sutures, titanium mesh and neuro-patch) were removed; Second, rinsed with large amount of gentamycin saline, betadine saline and hydrogen peroxide; Third, the surgical instruments and surgical films were replaced, and then sutured by layers. **G**, **H** Representative photograph of isolation of *S. marcescens* from brain pus of the case

However, when we planned to remove the secondary sutures around the poorly healing wound on June 27, 2022, a copious amount of a thin yellow liquid was squeezed out of the wound. ﻿It was then contemplated that titanium implants should be removed immediately after performing another MRI examination. The imaging findings showed an evident light-yellow empyema around the titanium plate (Fig. 4).

**Fig. 5:**
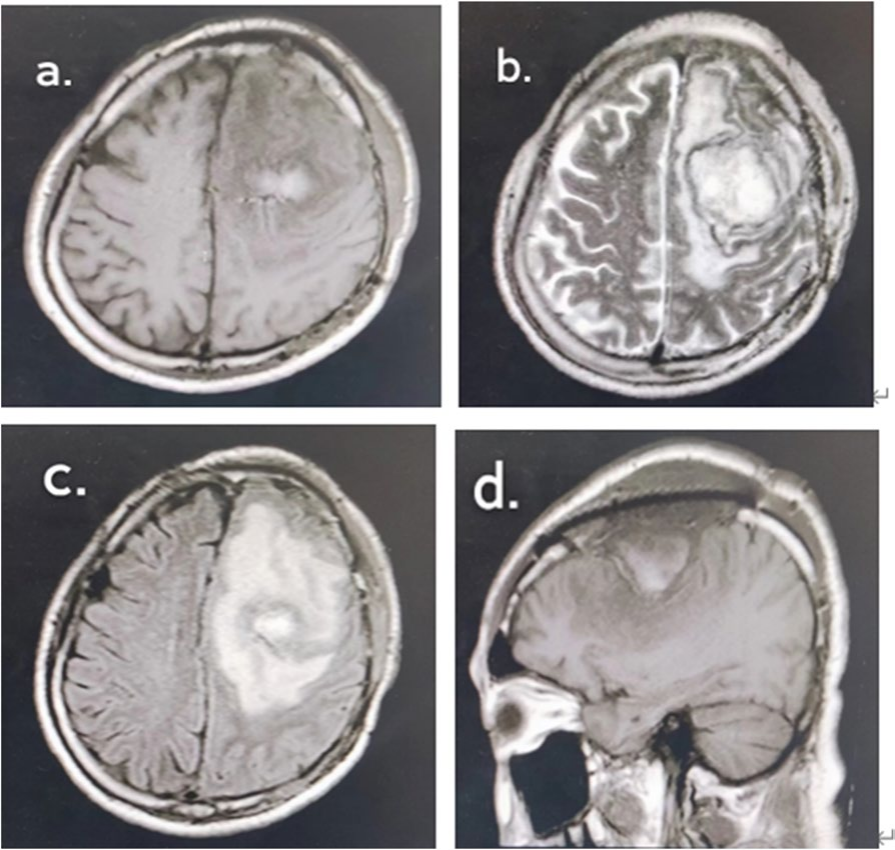
**a**-**d** Irregular equal-length mixed T1 inhomogeneous long T2 signal was seen in the left frontal lobe, and a short T2 signal ring was seen at the edge, with a cross-sectional size of about 46 mm × 46 mm × 40 mm, and a large cerebral edema was seen in the surrounding area

We reviewed the postoperative cranial MR on June 28, 2022. Cranial MR suggests postoperative changes in the left frontoparietal tumor, a high likelihood of a left frontal lobe hematoma, and a high likelihood of bilateral parietal subcutaneous hematomas or effusions (Fig. 5).

**Fig. 6:**
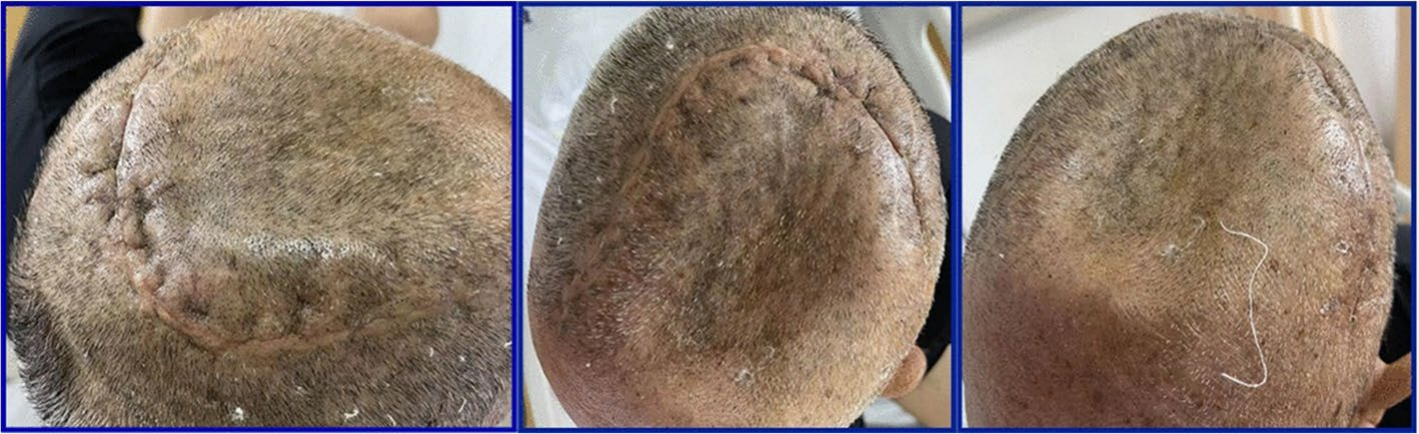
Patient's scalp fully healed at discharge

Surgical repair and debridement were performed by removing the titanium plate and cleaning up the suppurative secretions. Before discharge, five laboratory tests were conducted to identify possible pathogenic microorganisms. ﻿The first ﻿bacterial culture was obtained from wound discharge and the other three organisms were isolated from soft tissues obtained during the surgical procedure. Only *Serratia marcescens* was isolated from all four cultures (Fig. 4G), which was identified to be not antibiotic-resistant. On the 10th day after brain debridement, the sutures were removed and the wound healed well (Fig. 6). After the patient was treated with sensitive antibiotics based on the drug susceptibility test results, the condition gradually improved, and he was transferred to a rehabilitation facility.

## Literature review and discussion

Herein, we present a unique case of an uncommon occurrence in which a 63-year-old male patient with a mass in the left parietal region developed an intracranial abscess caused by Serratia marcescens following surgery. Through this case, we would like to discuss several problems as follows. When and how to make hair preparation before surgery? How to quickly identify postoperative intracranial infection? We should be wary of Serratia marcescens in neurosurgery clinical work. How to deal with the challenges posed by infection after titanium mesh implantation.

In this case, the patient's preoperative preparation was to shave his head the day before the operation instead of cutting hair. Whether ﻿the presence of hair at surgical sites increases the risk of neurosurgical infections remains controversial. Preoperative head shaving improves the visibility of the incision line and reduces postoperative surgical site infections. However, many recent studies have reported that shaving the head can change the normal flora around the surgical area and usually results in minor trauma to the scalp, and both of which increase the risk of neurosurgical infection [14–25].

NICE guidelines identified that surgeons should opt to use clippers rather than shaving for hair removal [26]. Razors cause microtrauma, facilitating microbial entry and proliferation in incisions. Studies indicate increased SSIs with shaving versus clipping [27, 28]. Interestingly, few studies have evaluated the risk of SSIs between hair clipping and no hair removal. Forensic evidence shows individual-specific bacterial flora in hair, highlighting endogenous flora as a primary SSI source [29–31]. The necessity and efficacy of hair removal in preventing SSIs are debatable, as are the effectiveness of disinfection methods for unshaved hair and strategies to mitigate clipping-associated risks, such as optimal timing and location of hair removal.

*Serratia marcescens* is a gram-negative bacterium that has garnered attention due to its association with healthcare-associated infections [10]. Serratia marcescens, commonly found in hospital environments, medical equipment, and on healthcare workers' hands, can infect various clinical samples like sputum, urine, and blood. Although most skin flora are gram-positive bacteria, Serratia marcescens infections have been reported in both healthy and immunocompromised individuals. *Serratia marcescens* has exhibited increasing resistance to multiple antibiotics, including β-lactams, aminoglycosides, and fluoroquinolones [32–34]. Its resistance mechanisms mainly include efflux pumps, downregulation or alteration of outer membrane pore proteins, chromosomally encoded antibiotic-modifying enzymes, plasmid-encoded ribosomal methyltransferases, and aminoglycoside-modifying enzymes [32].

Recent studies have focused on understanding the mechanisms of resistance and exploring alternative treatment options [32]. Combination therapies, such as the use of β-lactamase inhibitors in combination with β-lactam antibiotics, have shown efficacy in combating resistant strains [35, 36]. Additionally, research is underway to investigate the potential of novel antimicrobial agents and therapeutic approaches like phage therapy [37, 38]. Genomic analysis of *Serratia marcescens* provides a comprehensive understanding of its genetic composition, including virulence factors, antibiotic resistance genes, and regulatory networks [39–41]. It facilitates the identification of potential targets for therapeutic interventions, the development of diagnostic tools, and the implementation of effective strategies for controlling infections caused by this bacterium.

Antibiotics, the behind-the-scenes heroes of modern medicine, are used to treat various infectious diseases. However, several bacteria have become resistant to most currently available antibiotics. Staphylococci and drug-resistant gram-negative bacilli are the most likely causes of post-neurosurgical intracranial infections [42, 43]. The mortality rate of intracranial infections caused by gram-negative bacteria is significantly higher than that caused by other pathogenic bacteria [44]. Drug susceptibility tests indicated that only a few drugs, such as polymyxins and aminoglycosides, were effective against gram-negative bacteria. However, conventional drug delivery routes have low brain penetration rates, making it difficult to achieve effective therapeutic results. Thus, it is suggested to try some new routes of drug delivery such as intrathecal and intracerebroventricular administration [45]. In addition, the optimal antibiotic treatment for S. marcescens CNS infections remains controversial.

Currently, no clinical or preclinical studies demonstrate a direct relationship between the staging of meningiomas and infection. The pathology report after the patient's surgery showed that he had an angiomatous meningioma, which is a type of brain tumor classified as WHO I. Serratia marcescens infection occurred after surgery, possibly due to intraoperative contamination or suboptimal preoperative shaving, and was not significantly associated with the meningioma stage. We don't detect this latent infection very quickly. Therefore, exploring some methods for rapid identification of postoperative intracranial infection is necessary.

Some cytokines can be used as markers to diagnose bacterial infections. Significantly elevated levels of cerebrospinal fluid IL-6, IL-8, and INF-α occur in bacterial intracranial infections. Cerebrospinal fluid (CSF) analysis is currently considered the gold standard for diagnosing intracranial infections, and biomarkers such as CSF lactate, heparin-binding protein, C-reactive protein, and procalcitonin play ancillary roles [46–49]. With the development of molecular biology, laboratory diagnostic tools have evolved from the cellular to the molecular DNA levels [50, 51]. Pathogenic high-throughput genetic testing enables the rapid detection of disease-causing microorganisms. In addition, diffusion-weighted MR imaging (DWI) and apparent diffusion coefficient (ADC) help distinguish abscesses from other pathologies.

Infection of titanium mesh implants is a common postcranioplasty complication. We analyzed the various causes of titanium implant-associated infections after cranioplasty, such as poor underlying conditions of patients, failure to achieve strict aseptic operation or contamination of the titanium mesh during the procedure, poor local blood supply due to pressure on the scalp caused by a poorly shaped titanium mesh, and local tissue necrosis due to excessive intraoperative hemostasis [52, 53]. Second, cranioplasty requires breakthroughs in materials science, and finding new alternative biomaterials is an urgent clinical requirement. Recent studies revealed that PEEK has the lowest risk of cranioplasty revision and new hydrogel biomaterials show promising antibacterial properties and bone regeneration effects [54–57]. In this case, the autogenous bone was discarded after the meningioma was found to erode the skull, and a titanium mesh was selected for repair during the craniotomy procedure. However, it is questionable to rashly use allogeneic metal materials for repair when the grade of meningioma is not clear enough.

## Conclusion

Serratia marcescens is a rare but significant cause of postoperative brain abscesses, often leading to high mortality despite advanced antibiotics and radiology. This pathogen, not previously reported in adult neurosurgery, poses a significant risk for nosocomial infections. The case and literature review aims to share insights for diagnosing and treating such infections.

Preventive measures include proper hair removal before surgery, monitoring postoperative inflammatory markers, and early diagnostic imaging for delayed wound healing. Effective treatment hinges on selecting appropriate antibiotics based on drug sensitivity and understanding their mechanisms. New therapies, including phage therapy and antibiotic combinations, are promising against this pathogen. Additionally, correct titanium mesh implantation and stringent sterilization are crucial to prevent related infections, with research on infection-resistant materials ongoing. Ultimately, timely removal of artificial materials and abscess resection are key to managing these infections.

## Data Availability

If someone wants to request the data and materials from this study, they should contact Wenzheng Liu/lwzdoct@163.com.
